# Single point motion kinematics convey emotional signals in children and adults

**DOI:** 10.1371/journal.pone.0301896

**Published:** 2024-04-10

**Authors:** Elisa Roberti, Chiara Turati, Rossana Actis-Grosso

**Affiliations:** 1 Psychology Department, University of Milano–Bicocca, Milan, Italy; 2 Neuromi, Milan Center for Neuroscience, Milan, Italy; University Hospitals Tubingen: Universitatsklinikum Tubingen, GERMANY

## Abstract

This study investigates whether humans recognize different emotions conveyed only by the kinematics of a single moving geometrical shape and how this competence unfolds during development, from childhood to adulthood. To this aim, animations in which a shape moved according to happy, fearful, or neutral cartoons were shown, in a forced-choice paradigm, to 7- and 10-year-old children and adults. Accuracy and response times were recorded, and the movement of the mouse while the participants selected a response was tracked. Results showed that 10-year-old children and adults recognize happiness and fear when conveyed solely by different kinematics, with an advantage for fearful stimuli. Fearful stimuli were also accurately identified at 7-year-olds, together with neutral stimuli, while, at this age, the accuracy for happiness was not significantly different than chance. Overall, results demonstrates that emotions can be identified by a single point motion alone during both childhood and adulthood. Moreover, motion contributes in various measures to the comprehension of emotions, with fear recognized earlier in development and more readily even later on, when all emotions are accurately labeled.

## Introduction

The contribution of motion to the recognition of emotional expressions has been questioned for decades. Many studies have demonstrated that dynamic cues could facilitate the recognition of facial emotional expressions in adults [1, 2], children [3], and even infants [4]. Furthermore, the kinematic component is crucial when one considers emotions as expressed with "body language": hands, posture, and gait contribute widely to emotion expression, recognition, and comprehension [5].

A traditional way used to investigate the contribution of kinematics to perception and cognition takes advantage of the so-called point-light displays (PLDs) [6]. PLDs technique relies on applying illuminated markers placed on some crucial points (i.e., joints) of a human body, while all other body features are masked. When presented as static, the PLD is not recognized as a meaningful configuration, but as soon as the markers are moving, from birth we immediately recognize the movement of a human body [7–9]. PLDs have been used to separate information concerning motion from any other type of visual information [8, 10]. As humans’ and animals’ locomotion [11] is readily available from PLDs, they are often referred to as "biological motion." Research on bodily emotions [12] shows that children and adults can categorize emotions when expressed with static body posture, but with a far lower accuracy as compared to dynamic stimuli [3, 13–15]. Thus, while recognizing facial emotional expressions may be possible with static and moving images [16, 17], this is not true with other body parts, which rely heavily on kinematic cues to express emotions. Research on bodily expressions of emotions has thus primarily focused on *moving* bodies [13, 18, 19], with plenty of studies using the PLD technique with adults [20–23], children [15], and infants [24], showing that PLDs are sufficient for the perception of emotions.

However, several authors [18, 25] have pointed out that the PLDs technique does not separate the kinematic component from configural information: some information related to the shape could be extracted even from PLDs due to the motion coherence of the visible points, which determines the vivid impression of a moving figure. This is a very well-known effect in psychology reported as common fate [26], and also as structure-from-motion perception [27] or shape-from-motion perception [28]. Thus, the perceptual system could extract the same structural components available in static pictures from the moving pattern. Following this line of reasoning, it is unsurprising that dynamic emotional stimuli are better recognized than static ones, given that more information is provided to the perceptual system.

To sum up, the challenge of understanding the relevance of the kinematic component in emotion recognition remains, given that the kinematic, configural, and featural information are all present in dynamic emotional stimuli, PLDs included.

We reasoned that a possible way to solve this puzzle would be to study the recognition of emotions conveyed by a single point. This idea stems from studies investigating biological motion from the perspective of the motor theory of perception [29–31], which demonstrated that a "biological kinematics" exists independently from any configural information. Rather, the process of perceptual selection is constrained by the implicit knowledge that the central nervous system has depending on the movements it can produce. In other words, our perceptual system is very well attuned to a peculiarity of human movement, namely a specific relation between velocity and curvature (i.e., the two-thirds power law) [32, 33], which is thus a low-level description of biological motion. The sensitivity to biological motion of a single point-of-light has been investigated in adults [31, 34–36]. Even 4-day-old newborns look longer at the non-biological motion, suggesting that the movement in which the two-third-power law is not respected violates their expectations [37].

Similar reasoning also applies to classical studies on animacy [38, 39]. In those studies, by showing a specific dynamic display, authors demonstrated that social perception on the one hand [38] and causality on the other [38, 39] are stimulus-driven and strictly related to the kinematics of the displayed animation. Later on, studies on animacy [40, 41] typically showed geometrical forms moving according to specific kinematics and dynamics, providing important insights into the foundations of perceptual processes involved in our understanding of interpersonal dynamics and social signals.

In the present study, in analogy with both the two-third power law and animacy studies, we wanted to verify whether it is possible to recognize a specific emotion (namely fear, or joy) as conveyed only by kinematics (i.e., absolute velocity, accelerations/decelerations, stops, etc.) and dynamics (i.e., the "form" of the trajectory, wavelike, parabolic, rectilinear, etc.). In other words, we sought to determine whether it is possible to identify the specific law of motion related to particular emotions, from which children and adults could perceive a single point of light (or a meaningless geometrical form) as happier or sadder. In this way, the link between emotions and motion perception throughout development could be clarified. The comparison between adults’ and children’s performance could help us better understand the origins and development of emotion recognition from motion. Indeed, while a large amount of literature is dedicated to the investigation of the development of infants’ [42] and children’s [43] ability to recognize emotional expressions and some studies have addressed this issue using moving faces [13] or body movements [15], none has ever investigated whether the kinematic of a single point/geometrical form is capable of conveying an emotional signal.

We chose to create emotional motion patterns by replicating the kinematics and dynamics (which will be detailed in the Stimuli Section) from cartoons in which the character displayed a fearful or a happy emotion. It should be noticed that in cartoons, emotions tend to be magnified in the same way human actors adopt when conveying different emotions only with the body [13]. Following this approach, it was possible to convey happy and fearful emotional signals by manipulating the motion of a *single* and meaningless point/geometrical form, thus avoiding any possible confounding with other configural or featural elements of the display. We created three motion patterns conveying happiness, fear, and a neutral state. Interestingly, these patterns nicely matched Chafi and colleagues [44], where three specific motion patterns (i.e., Translational, Parabolic, and Wavelike) have been associated with different emotions displayed by faces. The present study conveyed fear through parabolic and happiness through wavelike trajectories. Translational trajectories were used for neutral animations (i.e., not conveying emotional expression). In a forced-choice paradigm, these animations were shown to a group of adults, along with two groups of children. Specifically, we included children aged 7 and 10 years, documented in the literature as significant turning points in which accuracy in identifying emotional displays portrayed by full-light and point-light video clips increases significantly [15, 45]. We expect the group of adults to be able to associate each pattern of motion correctly and quickly (as indexed by accuracy and reaction times) with the corresponding emotion. The observed performance at different ages could help us better understand whether movement’s contribution in emotion recognition might change throughout development.

Lower reaction times and higher accuracy for a specific emotional category would indicate a preferential processing of that category. We also measured two other indices used to measure response competition: the maximum deviation (MD) and area-under-the-curve (AUC) of the motor response given with the mouse. Mouse tracking paradigms, employed both with adults [46] and children [47], are based on the idea that hand motions may reflect a continuous motor trace of the tentative to commit to a specific behavioral choice [48–50], and thus these measures reflect real-time decision-making and how this changes motor programs. A comparison between the ideal trajectory (i.e., the straight line from the initiation to completion points) and the actual trajectory is indexed by the largest perpendicular deviation between the mouse movement and the straight trajectory (MD) and the geometric area between the mouse movement and the straight trajectory (AUD). As such, this measure would detect whether some motion patterns, and thus some real-time choices, are more easily identified than others.

To date, no study specifically investigated the possibility that emotions could be recognized when conveyed only by the motion of a single point/geometrical form. The present study poses itself as exploratory and seeks to take the first steps in investigating how emotions can be transposed purely into kinematics.

## Material and methods

### Participants

Data was collected between September 28 and October 30, 2018. The sample included in the present study consisted of 30 adults (mean age = 25.78 years, SD = 4.35 years, 9 males), 30 10-year-olds (mean age = 9.69 years, SD = 4.15 months, 16 males), and 30 7-year-olds (mean age = 7.31 years, SD = 3.78 months, 14 males). An additional 7-year-old child was tested but excluded from the sample as he did not complete the procedure. A power analysis was performed prior to conducting the study. We chose to aim for a moderate effect as the smallest detectable effect, setting the partial eta squared of 0.06 as an effect size estimate [51]. Using MorePower [58] software, we calculated the sample size for the interaction between the three emotional categories and the three age groups and two genders [52] with repeated-measures ANOVA. Setting alpha at 0.05 (two-sided), partial eta squared ($\eta_{p}^{2}$) at 0.06, and a power of 1- β = 0.80, the software yielded an optimal sample size of 90 participants in total. Adult participants were students recruited from the University of Milano–Bicocca. Child participants were recruited in the suburban areas of Milano and Lecco in northern Italy, and the teachers reported them not to have any history of a neurological or significant medical condition. All participants had normal or corrected to normal vision.

Before the testing sessions, all adult participants and parents gave their written informed consent, while verbal consent was obtained for the 7- and 10-year-olds, according to the ethical standards of the Declaration of Helsinki (BMJ 1991; 302:1194). The ethics committee of the University of Milano—Bicocca approved the study on September 21, 2018 (protocol n. 395). Data have been anonymized so that no individual participants can be identified after data collection.

### Stimuli

The stimuli consisted of videos in which a single animated geometrical shape on a black background moved, conveying a happy, fearful, or neutral emotion. The neutral animation followed a Translational trajectory to represent a movement that would not be associated with any emotional expression. The emotional animations were created from a selection of cartoons in which the character (e.g., Tom & Jerry) displayed a fearful or a happy emotion through a Wavelike body movement for the positive emotion and a Parabolic trajectory for the negative emotion. Individual frames were extracted using Virtual Dub 1.9.11 (http://www.virtualdub.org/) and imported into a Microsoft PowerPoint 97–2003 presentation. A geometrical form was added to each frame and aligned to the top-left point in the character’s body, which was then removed from the scene. This allowed preserving only the cues about movement in space, while all other pictorial emotional information (i.e., facial expressions and posture) were removed. To make the task more interesting and diverse for children, the moving geometrical form expressing each emotion (happy, fearful, and neutral) was presented in 3 different shapes (circle, square, triangle), 2 different colors (white, yellow), and could start its movement from the two sides of the screen (left, right), for a total of 36 videos. The square’s sides were 128 pixels (4.52 cm), the triangle’s sides were 126 (4.45 cm), 140 (4.94 cm), and 140 (4.94 cm) (height: 124 pixels, 4.37 cm) pixels, and the circle had a diameter of 126.81 pixels (4.48 cm). The luminance of the videos was checked with a Minolta CS-100 photometer for the two colors presented. The yellow animation had a luminance of 90.4 cd/m^2^, and the white animation had a 108 cd/m2 luminance.

**Fig 1:**
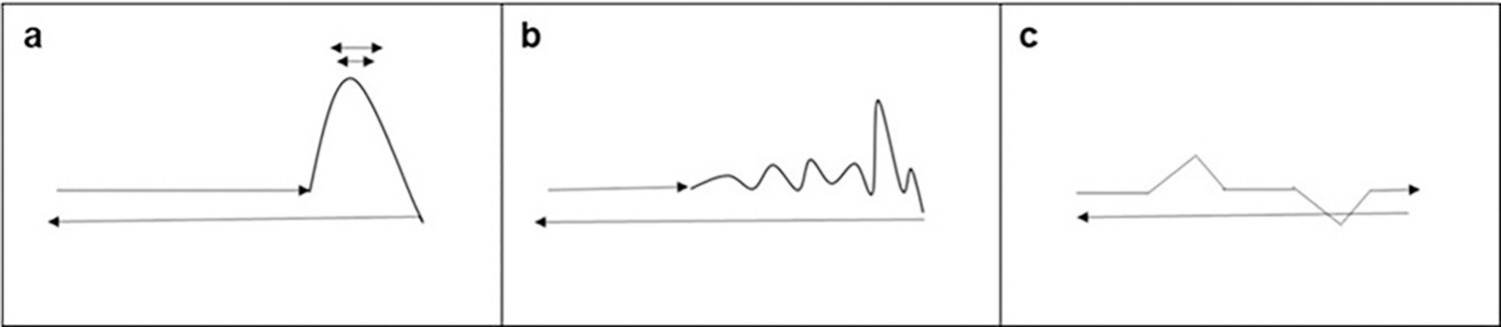
Trajectories for the fearful **(a)**, happy **(b)**, and neutral **(c)** stimuli. (a) After a Translational entrance, the shape jumps, shakes, and goes back down following a Parabolic course; (b) the shape jumps following a Wavelike motion; (c) a neutral Translational series of movements.

All three movements started with an initial entry motion that followed a linear path and ended with a final backward motion, in which, after reaching three-quarters of the screen, the geometrical shape turned around and returned from the same path. The total length of each video was of 3 seconds. *(a) Fearful motion* (Fig 1, panel a). After an entry motion of 861 pixels, the shape "jumped" (with a parabolic trajectory) and started "shaking." The jump started from a velocity of 0.14 m/s (the same as the entry motion) and had a linear acceleration until it reached the top of the vertical trajectory (184.6 pixels, 6.53 cm in height). At the top of the jump, the square moved back-and-forth along a small horizontal trajectory of 139.4 pixels (4.9 cm) (i.e., "shaking" behavior) five times. Then it moved down to go back along a horizontal trajectory of 861 pixels (30.4 cm), with a higher velocity (i.e., 0.24 m/s) and a constant acceleration. *(b) Happy motion* (Fig 1, panel b). After an entry motion of 664 pixels (23.42 cm), the shape started "jumping" in five jumps, following a wavelike trajectory. Each jump started from a velocity of 0.17 m/s (the same as the entry motion) and had a positive linear acceleration until it reached the top of the vertical trajectory, 82 pixels (2.89 cm) in height for the first jump, then 158.2 pixels (5.57 cm), 113 pixels (3.99 cm), 219.7 pixels (7.76 cm) and 141.8 pixels (5.01 cm) respectively) and a negative linear acceleration when it went down. After the jumps, the shape moved backward, following a linear path of 764.7 pixels (27 cm) with a constant velocity of 0.19 m/s. (c) *Neutral* video (Fig 1, panel c). After an entry motion of 748 pixels (26.39 cm), the shape went upward along a 65-degree tilted trajectory. It then went down and moved for 403.9 pixels (14.25 cm) to start an "inverse" jump (i.e., downward first), after which it went along a small horizontal path of 231 pixels (8.15 cm) and turned backward along a flat path of 527.3 pixels (18.6 cm). The whole motion had a constant velocity of 0.16 m/s. All videos are available at the following link: https://www.doi.org/10.17605/OSF.IO/8DGMW.

**Fig 2:**
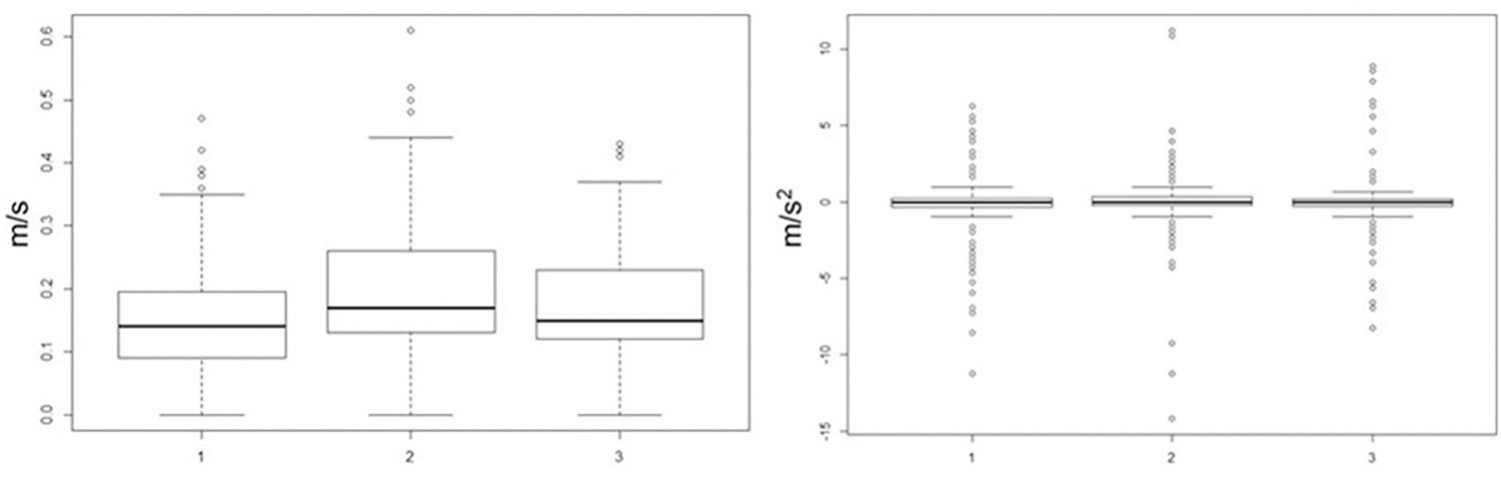
Boxplot for speed and acceleration patterns. The speed distributions are shown on the left, and the acceleration distributions on the right for neutral (1), fearful (2), and happy (3) videos.

A Mann-Whitney test also showed that the distribution of values between the angular acceleration values in the three videos did not differ (all p>.72) (Fig 2). Therefore, on average, no video contained more accelerating or slowing down movements than the others (mean acceleration: neutral: -0.24 m/s^2^; fear: -0.03 m/s^2^; happiness: 0.11 m/s^2^). The choice to present only happiness and fear as emotional stimuli is based on the fact that these two emotions are the earliest that children are able to recognize accurately [53, 54]. Furthermore, happiness is typically the easiest emotion to recognize by the movement of faces [61], but also with moving PLDs by adults, who rely more on moving PLD bodies than static faces when recognizing fear [55]. By choosing happiness and fear, we thus maximized the possibility of observing possible differences between different emotions as conveyed by pure motion.

### Design and procedure

**Fig 3:**
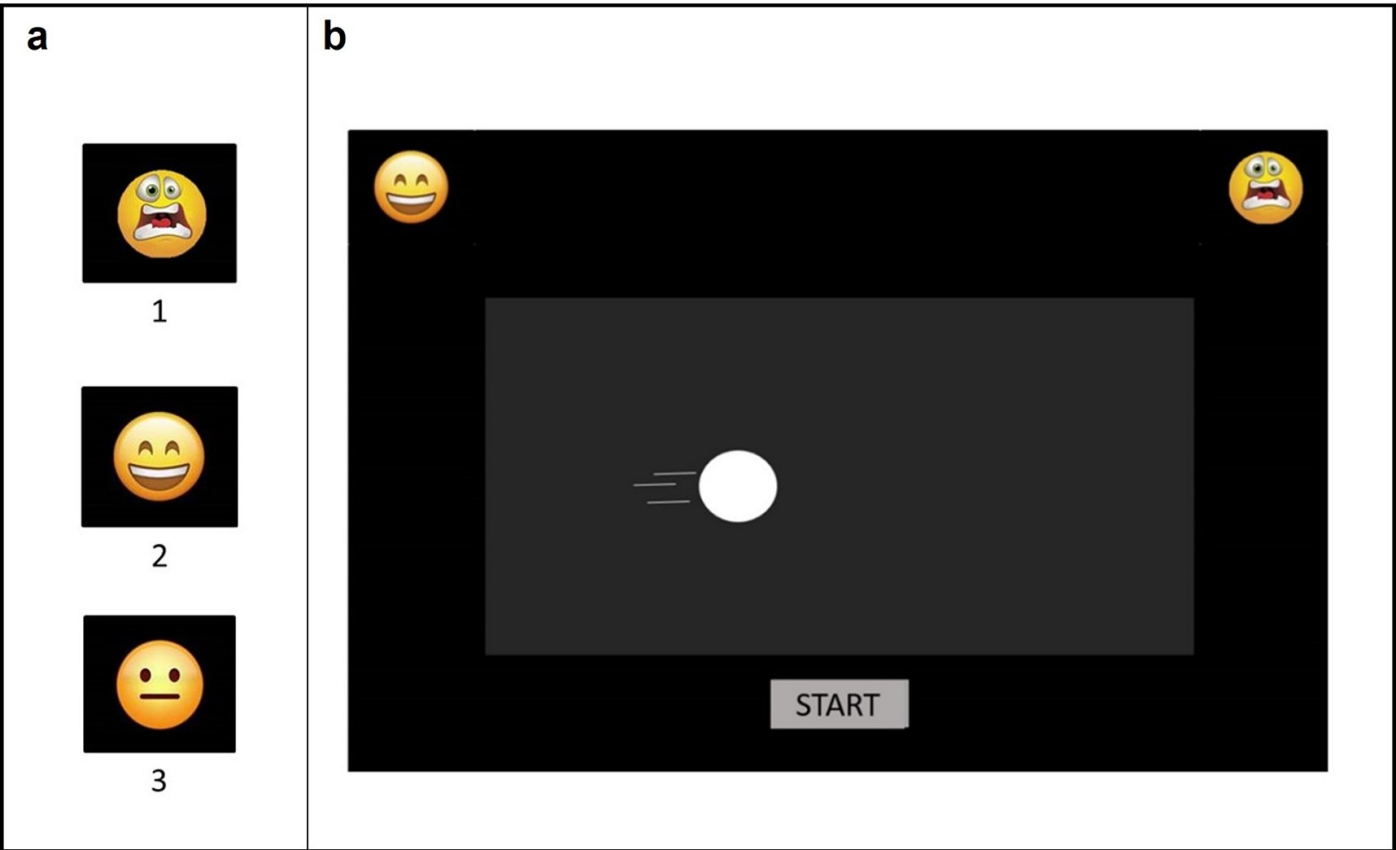
**(a)** Displayed key responses for the fearful (1), happy (2), and neutral (3) conditions. All the response buttons had the same dimension (1.43 x 1.29 cm) and were aligned to the top left and right corners of the screen. **(b)** Visual display of the experimental procedure, presented with Mouse Tracker. Participants pressed the "Start" button, and the videos appeared at the center of the screen. They were instructed to press the response button that better represented the emotional category of the stimuli by clicking a mouse button. After the response was given, another "Start" button appeared to begin the subsequent trial. No feedback on the accuracy was given to the participants.

Participants sat on a chair in front of a desk at a distance of 75 cm to the computer screen. They were told that they would see some short videos of geometrical shapes. The task required categorizing each video as quickly and accurately as possible by clicking with the mouse on one out of two emoticons (Fig 3, panel a) displayed at the computer screen’s upper left and right corners. The emoticons could be happy and neutral, happy and fearful, fearful and neutral, counterbalanced within participants as their left/right corner position. Six practice trials were administered before starting the experimental session. To begin each trial, participants had to press a "Start" button at the bottom-center of the screen, followed by the videos presented centered on the screen (Fig 3, panel b). The order of video presentation was randomized, and the stimuli were organized into three blocks of 12 videos each to allow children to take breaks if needed. All participants completed all three blocks, for a total of 36 trials, on average in 7 minutes (*SD* = 1.24 min). A Dell computer with a 15.6-inch screen connected via USB to a mouse was used for data collection. The Mouse Tracker software [48] was used. The mouse speed was set to the middle setting of Windows 7 [56].

### Data analysis

Accuracy was calculated for all age groups by dividing the number of correct answers by the total of the presented trials for each condition (i.e., happiness, fear, neutral). Reaction times (RT) in milliseconds, starting from the onset of the videos to the moment when a response was given, were recorded by the mouse-tracker and analyzed. From the Mouse Tracker software, AUC and MD measures were also extracted, measuring the attraction towards the two alternative emoticons displayed in the top corners of the computer screen [48]. Data were checked for outliers in reaction times in the first instance, calculated as exceeding the two standard deviations from the mean criteria. The trials in which an outlier was found were excluded from further analyses (trials per subject in the adult sample: *M* = 0.88; *SD* = 1.00; in the 10-year-old sample: *M* = 0.66; *SD* = 0.97; in the 7-year-old sample: *M* = 0.44; *SD* = 0.78). Accuracy scores were then calculated for each participant and each category. One-sample t-tests were performed to ensure that accuracy scores significantly differed from a 50% chance of response.

After excluding trials in which an incorrect response was given or the reaction time exceeded the two standard deviations threshold, 27.8 trials (*SD* = 6.4) per participant were included in average in the final analyses for the adult sample, a mean of 24.7 trials (*SD* = 5.2) for the 10-year-olds sample, and a mean of 22.4 trials (*SD* = 5.9) for the 7-year-olds. Considering incorrect responses and outliers, a mean of 2.73 trials (*SD* = 3.09) was eliminated in the adult sample, a mean of 3.78 trials (*SD* = 2.78) in the 10-year-olds sample, and a mean of 4.53 trials (*SD* = 2.95) in the 7-year-olds sample.

A 2 x 3 x 2 repeated measures analysis of variance (ANOVA) was performed for each age group with color (white, yellow), shape (circle, square, triangle), and direction of the movement (left, right) as within-subject factors, to make sure that these factors did not affect the responses to the task. As expected, for all age groups, the only significant effect was emotion (*p* = 0.02), while no effect of color, shape, direction, and no interaction were found (all *p*>0.09). Therefore, these factors were collapsed for further analyses.

For each participant, a RT, MD, and AUC score was then calculated for the three emotions (happiness, fear, neutral). For each of the variables, the normality of distributions was checked with the Shapiro–Wilk normality test (RT: *p* < .001, MD: *p* < .001, AUC: *p* < .001). Separate ANOVAs were computed for the three dependent variables, with emotion as within-subject factor and age group (adults, 10-year-olds, 7-year-olds) as between-subject variable. Possible gender effects were also investigated, adding gender as a between-subject variable.

Pairwise t-test comparisons, where necessary, were conducted using a Bonferroni correction. The significance threshold was set at 0.05, and a Greenhouse-Geisser correction was applied whenever the assumption of Sphericity was violated (indicated by ε). Effect sizes were indicated with Cohen’s *d* for t tests and partial eta squared for ANOVAs [57, 58].

## Results

### Accuracy

In the adult sample, participants’ accuracy was significantly different than the 0.5 chance level for the happy (*M* = 0.83; *SD* = 0.20), *t* (29) = 7.57; *p* < .001, *d* = 1.20, fearful, (*M* = 1; *SD* = 0.29) *t* (29) = 6.56; *p* < .001, *d* = 1.38, and neutral condition (*M* = 0.76; *SD* = 0.24), *t* (29) = 6.13; *p* < .001, *d* = 1.12. In the 10-year-old sample, the same was found, with high accuracies for the happy (*M* = 0.70; *SD* = 0.20), *t* (29) = 5.43; *p* < .001, *d* = 1.21, fearful (*M* = 0.83; *SD* = 0.27), *t* (29) = 6.63; *p* < .001, *d* = 0.99, and neutral condition (*M* = 0.62; *SD* = 0.18), *t* (29) = 3.71; *p* < .001, *d* = 0.67. For the 7-year-olds, this was true for the fearful (*M* = 0.75; *SD* = 0.29), *t* (29) = 4.73; *p* < .001, *d* = 0.86, and neutral condition (*M* = 0.61; *SD* = 0.21), *t* (29) = 2.93; *p* = .007, *d* = 0.53, while for the happy condition, the accuracy was not significantly different than chance (*M* = 0.58; *SD* = 0.25), *t* (29) = 1.76; *p* = .089, *d* = 0.32.

Therefore, except for the happy stimuli in the 7-year-old group, the animations presented were accurately recognized at all ages, supporting the validity of the stimuli set toward communicating the expected emotional valence or absence thereof. A 3 x 3 x 2 repeated measures Analysis of Variance (rmANOVA) was performed with the three emotion categories as within-subjects factors and the three age groups and gender (male, female) as between-subject factors to check if the accuracy between ages and gender differed. As expected, there was a significant effect of emotion, *F* (2, 206) = 9.19, *p* < 0.001, $\eta_{p}^{2}$ = 0.08. Post-hoc tests showed that fear’s accuracy (*M* = 0.86; *SD* = 0.28) was higher than both the happy (*M* = 0.83; *SD* = 0.22) and the neutral accuracy (*M* = 0.66; *SD* = 0.21). An effect of age group was also found, *F* (2, 103)  =  11.7, *p* < 0.001, $\eta_{p}^{2}$ = 0.19). No main effect of gender or interactions with gender were found (all *p*>0.31). Post-hoc tests showed that adults’ accuracy (*M* = 0.86; *SD* = 0.24) was higher than both the 10-year-olds (*M* = 0.72; *SD* = 0.22) and the 7-year-olds (*M* = 0.65; *SD* = 0.25) and that 10-year-olds accuracy was higher than the one of 7-year-olds (all *p* < .04). No emotion by age interaction was observed in accuracy values (*p* = 0.29).

### Reaction time (RT)

**Fig 4:**
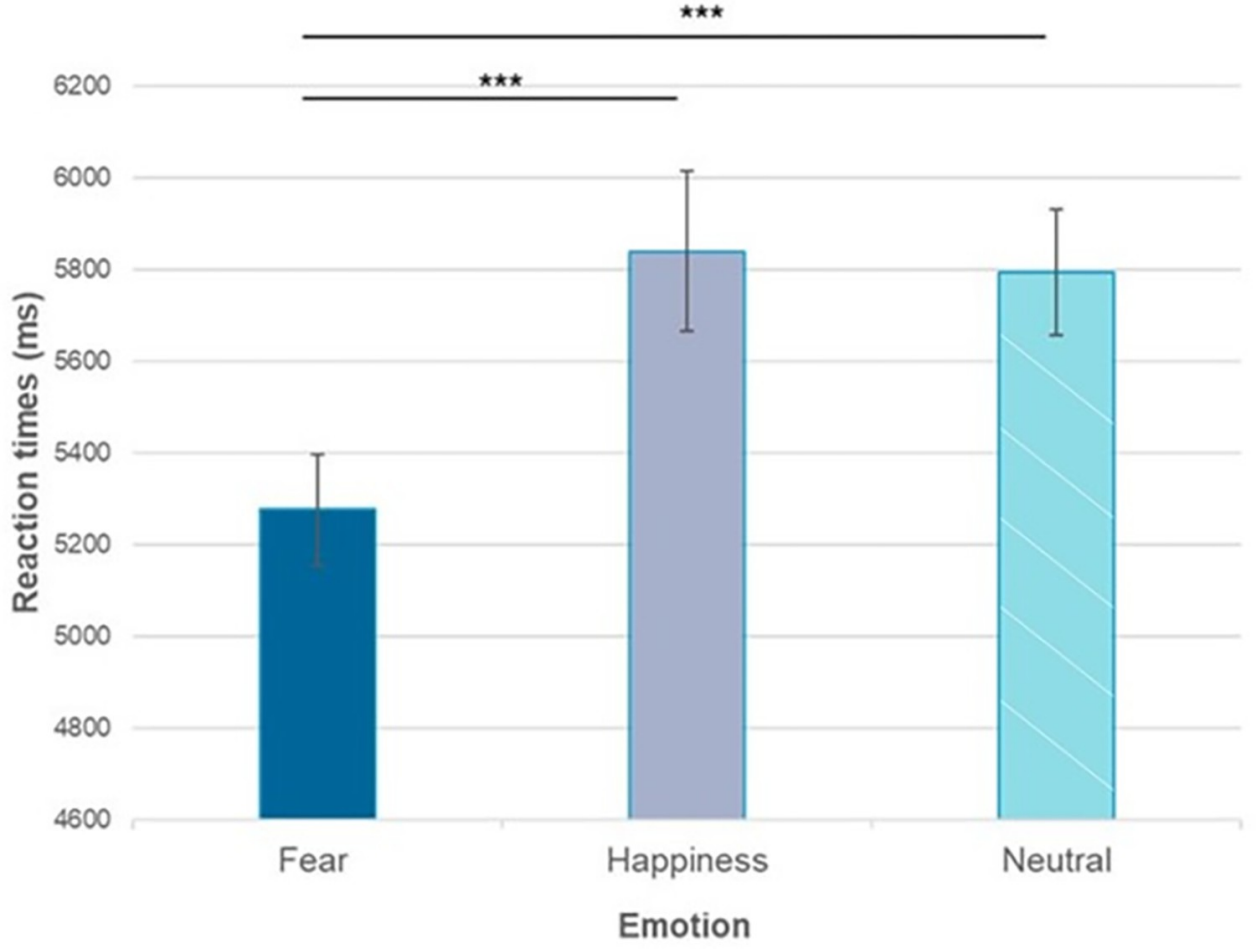
RTs observed for the different emotions across age groups. In the fearful condition, RTs were lower than in both the happy and the neutral conditions, while the happy and the neutral condition did not differ from each other (***p < 0.001). Error bars represent the standard errors of the means (dark grey: fear; light grey: happiness; diagonal lines: neutral).

First, we conducted an ANOVA with emotion (Fear, Happiness, Neutral) as within-subject factor and age group (adults, 10-year-olds, 7-year-olds) and gender (male, female) as between-subject variables. A main effect of emotion, *F* (1.44, 125.61)  =  17.25, *p* < 0.001, $\eta_{p}^{2}$ = 0.17, *ε*  =  0.72, and age group, *F* (2, 87)  =  58.5, *p* < 0.001, $\eta_{p}^{2}$ = 0.57, were observed. No main effect of gender or interactions with gender were found (all *p*>0.09). Therefore, t-test comparisons were carried on for the emotion and age group factors separately. Averaging among the three age groups, we observed that RTs in the fearful condition (*M* = 5276 ms; *SD* = 1151 ms) were lower than in both the happy (*M* = 5840 ms; *SD* = 1660 ms*; t* (29) = -4.26, *p* < .001, *d* = -0.78) and the neutral (*M* = 5794 ms; *SD* = 1304 ms; *t* (29) = -6.51, *p* < .001, *d* = -1.19) condition. The happy and the neutral condition, in contrast, did not differ from each other (*p* > 0.9) (Fig 4).

Averaging among the three emotions, the t-tests indicated that adults (*M* = 4561 ms; *SD* = 185 ms) were faster than 10-year-olds (*M* = 5482 ms; *SD* = 363 ms; *t* (29) = -5.27, *p* < .001, *d* = -0.96) and 7-year-olds (*M* = 6866 ms; *SD* = 420 ms; *t* (29) = -9.47, *p* < .001, *d* = -1.73), and 10-year-old children were also significantly faster than the 7-year-old children, (*t* (29) = -6.56, *p* < .001, *d* = -1.2).

**Fig 5:**
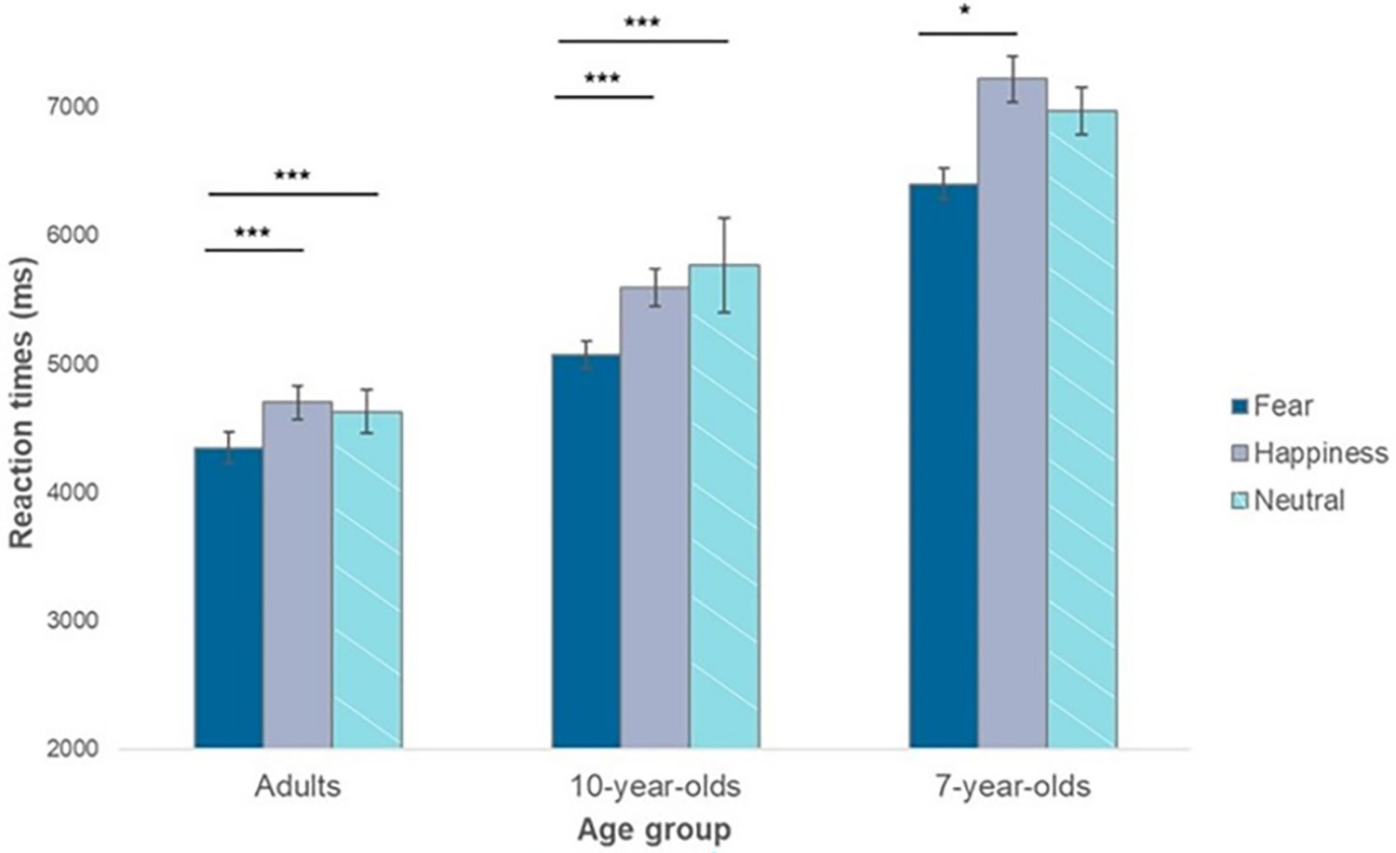
Results of the ANOVAs performed separately for the three age groups. In the adult and 10-year-olds groups, faster RTs in the fearful condition compared with the happy and the neutral condition. In the 7-year-olds, lower RTs were observed only for fear compared with happiness (***p < 0.001; **p < 0.01; *p < 0.05). Error bars represent the standard errors of the means (dark grey: fear; light grey: happiness; diagonal lines: neutral).

To further investigate the differences within the different age groups in terms of responses to emotions, three separate ANOVAs were carried out (Fig 5). In the adult group, the pattern of a main effect of emotion found in the general ANOVA was confirmed, *F* (2, 58)  =  8.66, *p* < 0.001, $\eta_{p}^{2}$ = 0.23, with lower RTs in the fearful (*M* = 4351 ms, *SD* = 662) condition compared with the happy (*M* = 4702 ms, *SD* = 599; *t* (29) = −3.93, *p* < 0.001, *d* = -0.71), and the neutral condition, (*M* = 4632 ms, *SD* = 662; *t* (29) = −3.15, *p* = 0.004, *d* = -0.58). The same pattern was found for 10-year-olds, *F* (2, 58)  =  25.5, *p* < 0.001, $\eta_{p}^{2}$ = 0.47, with lower RTs in the fearful condition (*M* = 5243 ms, *SD* = 768) compared with the happy, (*M* = 5684 ms, *SD* = 787; *t* (29) = −5.14, *p* < 0.001, *d* = -1.12), and the neutral condition, (*M* = 5868 ms, *SD* = 931; *t* (29) = −6.72, *p* < 0.001, d = - 1.23). For the 7-year-olds, although the general effect of emotion was still present, it had a small effect size, *F* (2, 58)  =  4.20, *p*  =  0.02, $\eta_{p}^{2}$ = 0.12, and the paired-sample t-test revealed that fear (*M* = 6588 ms, *SD* = 1206) only differed from happiness (*M* = 7040 ms, *SD* = 2043; *t* (29) = −2.82, *p* = 0.008, *d* = -0.4). Again, for all age groups no main effects of gender or interactions with gender were found (adults: all *p*>0.2; 10-year-olds: all *p*>0.4; 7-year-olds: all *p*>0.1).

### Area-under-the-curve (AUC) and maximum deviation (MD)

The ANOVA with AUC as a dependent variable revealed a significant interaction between emotion and age group, *F* (4, 174)  =  2.97, *p*  =  0.021, $\eta_{p}^{2}$ = 0.06. No main effect of gender or interactions with gender were found (all *p*>0.2). To further investigate this interaction, post-hoc comparisons were conducted and revealed that, for the 10-year-old sample, the area between the AUC was smaller for fearful (*M* = 0.37, *SD* = 0.5) than for neutral responses (*M* = 0.79, *SD* = 0.7; *t* (29) = −2.9, *p* = 0.007, *d* = -0.52).

**Fig 6:**
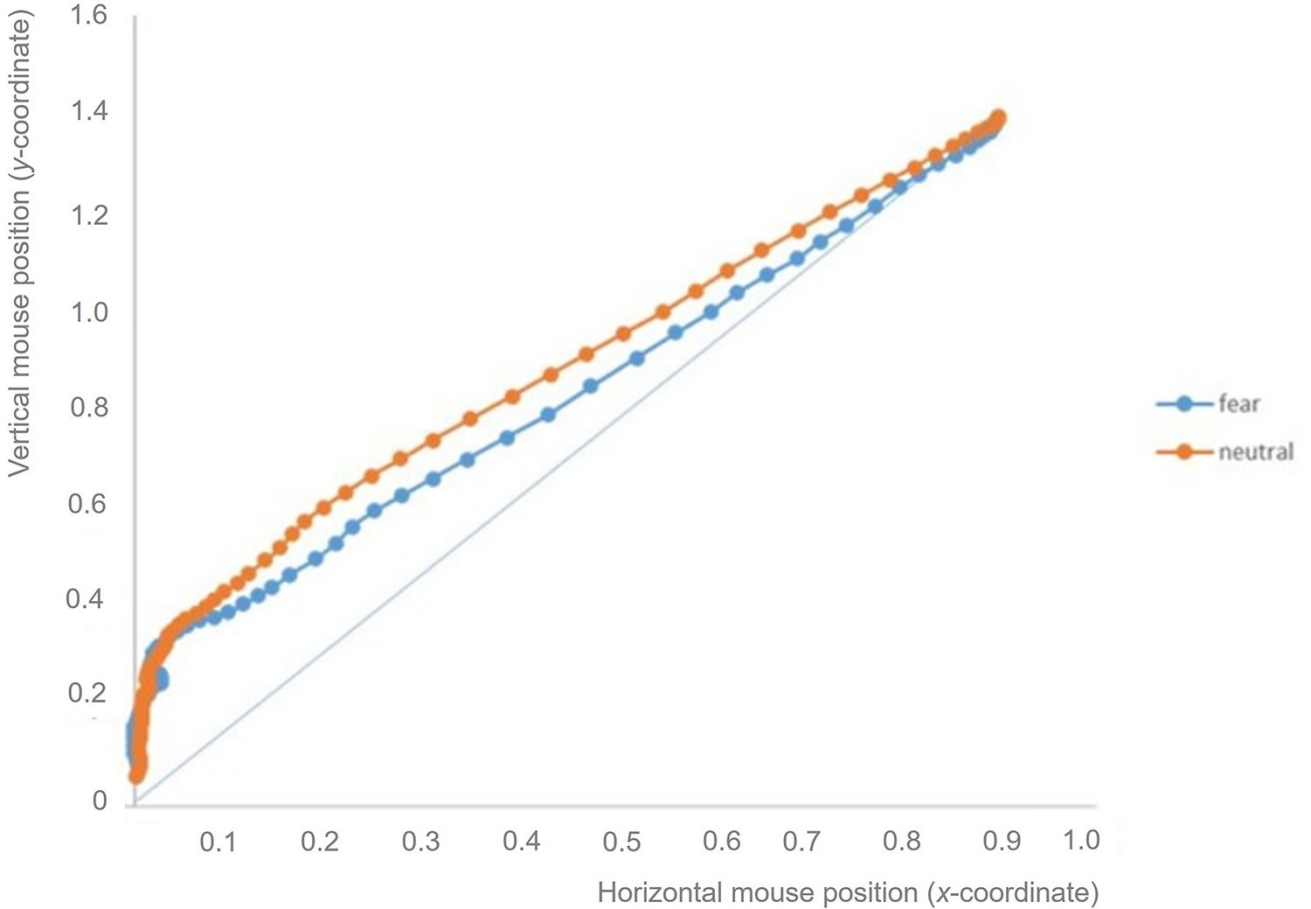
AUC and MD, Mouse Tracker indexes of the strength of attraction towards the alternative response for the 10-year-old children (the upper line represents responses to the neutral condition; the lower line represents responses to the fear condition).

A similar pattern was also evident when considering the MD measure. In this case, the main effect of emotion, *F* (2, 174)  =  3.86, *p*  =  0.023, $\eta_{p}^{2}$ = 0.04, was further qualified by the interaction between emotion and age group, *F* (4, 174)  =  4.11, *p*  =  0.003, $\eta_{p}^{2}$ = 0.09. No main effect of gender or interactions with gender were found (all *p*>0.4). Again, for the 10-year-olds, the largest perpendicular deviation of the mouse was smaller for fearful (*M* = 0.17, *SD* = 0.2) than for neutral responses, (*M* = 0.33, *SD* = 0.2; *t* (29) = −3.30, *p* = 0.003, *d* = -0.60 (Fig 6). The similarity in AUC and MD findings confirms that both measures reflect the same continuous unfolding of cognitive processes during the execution of behavioral responses [49].

## Discussion

This study tested the possibility that humans recognize different emotions as conveyed only by a single moving geometrical figure. Moreover, we sought to investigate whether this competence’s developmental onset and developmental trajectory are consistent across emotions or differ according to the specific emotion considered. Three age groups, 7-year-old children, 10-year-old children, and adults, were presented with a geometrical shape, which could move according to a happy, a fearful, or a neutral motion, and asked to categorize each animation in a forced-choice paradigm. The movement of the mouse while the participants’ selected response was also tracked.

Our main result shows that adults and 10-year-old children recognized happiness and fear when conveyed by the animations presented, while 7-year-olds could only recognize fear. This finding has important implications for future studies on emotion recognition, given that it highlights the perceptual system’s ability to extract information regarding emotion from single-point displays. The contribution of motion (from both face and body) to the recognition of emotions has been questioned for decades. Our results suggest that motion alone (without any figural confound) can also convey the emotion of fear and happiness.

We found that kinematics contributes in various measures to the comprehension of the different presented emotions. This consideration is supported by both the difference found in accuracy and RTs for adults and 10-year-old children and by the performance of 7-year-olds. In particular, for adults and 10-year-olds, fear’s accuracy was higher than for both happy and neutral stimuli. Fear was recognized faster than both happiness and neutral movement. At the same time, 7-year-olds could recognize only fear (and the neutral state), with the accuracy for the happy condition not significantly different from chance. Moreover, we found for 10-year-old children a straighter trajectory for fear identification in the mouse tracking variables. Given the assumption that hand motions reflect a continuous motor trace of a specific behavioral choice, this measure would index an easier identification for fearful stimuli. Taken together, these results suggest a *kinematic fear advantage*. Fear was well recognized by motion alone, while this was only partially true for happiness: we observed that the distinction between happiness and a neutral movement was more difficult at all ages (i.e., no differences between RTs for the happy and neutral condition), and 7-year-old children did not recognize happiness in our stimuli. As far as happiness is concerned, we might surmise that humans rely more on static/figural information present in happy faces (specifically, the smile) than dynamic cues, which do not appear to be particularly relevant to discriminate happiness from other affective states. This consideration is also based on the well-known happy face advantage [59], an effect often described in the scientific literature [60–62], for which happy faces are easier to discriminate than other emotional expressions even early in life [63].

The difference found in the present study between the perception of fearful and happy stimuli supports the idea that the static and the dynamic components of emotional expressions (conveyed by either faces or bodies) might be differently involved in recognizing different emotions [55]. This idea is nicely fitting an evolutionary perspective. Happiness (and its expression) is essentially aimed at increasing empathy [64] towards other individuals (i.e., conspecific). Consequently, no action is required when happiness is concerned, if not that of sharing some pleasant stimuli (such as food). Fear, on the other hand, as well as anger, communicates the presence of potential danger in the environment, and for this reason, being particularly relevant for survival is more likely to be conveyed by dynamic cues, which are not only more related to action but also more easily detectable from a distance (or when the other individual is not completely visible). In this perspective, fear and anger should be recognized more quickly. This idea is also supported by the fact that they are associated with increased vigilance, attention [65–67], and modulation of the motor system [68, 69]. Moreover, an advantage for fearful stimuli has been reported in several studies on recognition of emotional point-light displays both in typically developed individuals [13, 55, 70] and in individuals with Autistic Spectrum Disorder (ASD) [71–73]. Our results support the idea that this advantage is largely due to kinematics alone and not to the possible figural information extracted by PLDs’ motion coherence.

### Perspectives and limitations

Evidence accumulating is suggesting different processing for different emotions, particularly when fear and anger are considered [71, 74]. This supports the idea that natural selection resulted in a propensity to react more strongly to threatening stimuli [64].

Notably, being an exploratory study, only one sample of motion for each emotion was presented, adapted from cartoons. This approach might be criticized for not fully reflecting the natural kinematics of human bodies and for using a too-limited sampling of artificial kinematics.

Nonetheless, it is worth noticing that we did vary the color, shape, and direction of our animations. Specifically, the moving geometrical form expressing each emotion (happy, fearful, and neutral) was presented in three different shapes (circle, square, triangle), two different colors (white, yellow), and could start its movement from the two sides of the screen (left, right), for a total of 36 stimuli. Even more importantly, our choice was motivated by the aim of the study, which attempted to isolate the contribution of motion to emotion comprehension by relying upon magnified expressions of emotional signals conveyed through motion alone.

In this perspective, our study could be compared to the first studies on animacy [38, 39]. Like in those studies, it is not crucial to sample a range of movements (like Heider & Simmel, did not need to present various displays). In other words, we have to verify whether humans can recognize this relation without any "possible" form (human or human human-like). Thus, our crucial point was to show that human beings can recognize a specific emotion conveyed only by a single point’s motion. Future directions include investigating how this possibility could be generalized to all the movements representing different emotional contents.

Moreover, further studies could consider assessing the emotion recognition performance with gold-standard measurements, to correlate emotion comprehension from kinematics and other cues.

In our study, no gender differences were found. On the other hand, sex differences are often described in the literature when investigating the processing of emotional expressions through faces [75] or human biological motion [76]. While, for the present study, no specific hypotheses on gender effects were made, future studies might further investigate how gender differences impact the processing of kinematics of emotions throughout the lifespan.

Lastly, it should be noted that even though the mouse tracking measures are strongly related to motor skills, those measures complemented the results observed with accuracy and reaction times. Indeed, if it is true that accuracy and speed improve with age, the varying degree to which different stimuli are identified within the same age sample suggests that cognitive processes still play a fundamental role. Moreover, the mouse remains the most accessible tool for children aged 6 and older [77].

### Conclusion

To sum up, the present study constitutes the first evidence of the idea that different emotions can be extracted from the kinematics of movements alone across development. Further, this competence could be modulated by the specific emotion in the adult and the developmental population, where different developmental onsets were observed for happy and fearful kinematics. Indeed, in our sample fearful motion provided particularly rich information, quickly interpreted by all age groups. Happy motion was instead less readily categorized by adults and older children and poorly identified by our youngest participants. This developmental trajectory suggests that this ability could be improved with experience.
